# Supplementary material for: MetaRibo-Seq measures translation in microbiomes
Source: Nat Commun. 2020 Jun 29;11:3268. doi: 10.1038/s41467-020-17081-z (PMC7324362; doi:10.1038/s41467-020-17081-z)
Supplement: Supplementary file 10 — Supplementary Data 7 [file 41467_2020_17081_MOESM10_ESM.zip › File2/Confidence_High_Taxonomy/292534_out.krona.html]

Javascript must be enabled to view this page.

members
magnitude
magnitudeUnassigned
count
unassigned
taxon
rank

292534\_out

52

52
2
superkingdom

1239
52
phylum

class
52
186801

order
186802
52

family
52
186803


SRS013951\_contig\_number\_37014SRS013965\_contig\_number\_3699SRS014235\_contig\_number\_40758SRS014235\_contig\_number\_44092SRS014683\_contig\_number\_contig-100\_112.125157SRS015486\_contig\_number\_contig-100\_319.320SRS015579\_contig\_number\_contig-100\_920.155431SRS018427\_contig\_number\_contig-100\_1527.178562SRS018623\_contig\_number\_contig-100\_381.132510SRS019030\_contig\_number\_contig-100\_1111.120125SRS019161\_contig\_number\_43287SRS019601\_contig\_number\_contig-100\_825.166187SRS019685\_contig\_number\_23946SRS043701\_contig\_number\_contig-100\_595.118167SRS046717\_contig\_number\_contig-100\_1619.87464SRS049446\_contig\_number\_15566SRS049712\_contig\_number\_23919SRS050998\_contig\_number\_15044SRS052697\_contig\_number\_44215SRS056519\_contig\_number\_3098SRS056519\_contig\_number\_5986SRS063190\_contig\_number\_contig-100\_1216.100730SRS063370\_contig\_number\_contig-100\_178.88043SRS064276\_contig\_number\_42191SRS074586\_contig\_number\_604SRS077194\_contig\_number\_32971SRS077194\_contig\_number\_contig-100\_1088.193108SRS077641\_contig\_number\_contig-100\_315.136726SRS098073\_contig\_number\_18483SRS098644\_contig\_number\_48928SRS098644\_contig\_number\_contig-100\_864.280301SRS098717\_contig\_number\_16047SRS1041090\_contig\_number\_6717SRS1041133\_contig\_number\_14894SRS104400\_contig\_number\_55017SRS105153\_contig\_number\_contig-100\_326.229721SRS105153\_contig\_number\_contig-100\_618.230013SRS1055056\_contig\_number\_contig-100\_2948.61234SRS1055067\_contig\_number\_14519SRS142599\_contig\_number\_contig-100\_1053.189208SRS142890\_contig\_number\_41642SRS144362\_contig\_number\_39186SRS144362\_contig\_number\_44738SRS144537\_contig\_number\_40264SRS147425\_contig\_number\_contig-100\_197.85619SRS147977\_contig\_number\_contig-100\_602.87971SRS148424\_contig\_number\_contig-100\_1390.187012SRS148721\_contig\_number\_9671SRS149325\_contig\_number\_contig-100\_1560.96913SRS149784\_contig\_number\_5299SRS893252\_contig\_number\_contig-100\_714.118367SRS893378\_contig\_number\_contig-100\_764.137518
species
52
39491
